# Pulmonary Nontuberculous Mycobacteria–Associated Deaths, Ontario, Canada, 2001–2013

**DOI:** 10.3201/eid2303.161927

**Published:** 2017-03

**Authors:** Theodore K. Marras, Michael A. Campitelli, Hong Lu, Hannah Chung, Sarah K. Brode, Alex Marchand-Austin, Kevin L. Winthrop, Andrea S. Gershon, Jeffrey C. Kwong, Frances B. Jamieson

**Affiliations:** Mount Sinai Hospital, Toronto, Ontario, Canada (T.K. Marras, S.K. Brode);; University of Toronto, Toronto (T.K. Marras, S.K. Brode, A.S. Gershon, J.C. Kwong, F.B. Jamieson);; University Health Network, Toronto (T.K. Marras, S.K. Brode, J.C. Kwong);; Institute for Clinical Evaluative Sciences, Toronto (M.A. Campitelli, H. Lu, H. Chung, A.S. Gershon, J.C. Kwong);; West Park Healthcare Centre, Toronto (S.K. Brode);; Public Health Ontario, Toronto (A. Marchand-Austin, J.C. Kwong, F.B. Jamieson);; Oregon Health and Science University, Portland, Oregon, USA (K.L. Winthrop);; Sunnybrook Health Sciences Centre, Toronto (A.S. Gershon)

**Keywords:** Tuberculosis and other mycobacteria, mortality, Mycobacterium avium complex, Mycobacterium xenopi, Mycobacterium fortuitum, Mycobacterium abscessus, Mycobacterium kansasii, nontuberculous mycobacteria, survival, Ontario, Canada, bacteria

## Abstract

Survival implications of nontuberculous mycobacterial pulmonary disease (NTM-PD) and NTM pulmonary isolation without disease (NTM-PI) are unclear. To study deaths associated with NTM-PD and NTM-PI and differences in survival between them, we conducted a population-based cohort study of persons with microbiologically defined NTM-PD or NTM-PI diagnosed during 2001–2013 in Ontario, Canada. We used propensity score matching and Cox proportional hazards models to compare survival. Among 9,681 NTM-PD patients and 10,936 NTM-PI patients, 87% and 91%, respectively, were successfully matched with unexposed controls. Both NTM-PD and NTM-PI were associated with higher rates of death for all species combined and for most individual species. Compared with NTM-PI, NTM-PD was associated with higher death rates for all species combined, *Mycobacterium avium* complex, and *M. xenopi*. NTM-PD and NTM-PI were significantly associated with death, NTM-PD more so than NTM-PI.

Nontuberculous mycobacterial pulmonary disease (NTM-PD) is an increasingly common problem (*1*–*3*) that is associated with substantially impaired quality of life (*4*) and is difficult and costly to treat (*5*,*6*). At the population level, patients with NTM-PD have been poorly characterized in general, and their survival is not well studied. Studies from individual clinical programs have identified prognostic factors (*7*–*10*), but estimates of survival are undoubtedly affected by referral bias and therefore cannot be generalized to all NTM-PD patients. One population-based study of survival in NTM-PD patients has been reported, but it did not include controls without NTM, so NTM-attributable death could not be determined (*11*). Furthermore, differences in phenotypes between NTM-PD patients in Europe and North America (*12*) suggest that death rates might differ substantially between these regions. In the United States, a study of Medicare beneficiaries estimated a 40% higher risk for death in persons with NTM-PD than in persons without it, but the authors used a nonvalidated case definition and excluded patients <65 years of age and patients enrolled in health maintenance organizations (*3*).

In studying all residents of Ontario, Canada, who had NTM-PD, we sought to describe their clinical characteristics, estimate their survival, and determine whether patients with NTM-PD have higher rates of death than population age-, sex-, and propensity-matched unexposed controls. In addition, we sought to compare survival of patients with NTM pulmonary isolation but not disease (NTM-PI) with patients who had NTM-PD, as well as survival for patients with different NTM species.

## Methods

We conducted a population-based matched cohort study using linked health administrative data and mycobacteriology as described previously (*13*), including all Ontario residents with incident pulmonary NTM isolation diagnosed during 2001–2013, and unexposed controls matched by age, sex, index date, and propensity score (*14*). The responsible institutional review committees approved this study. Additional details about the data sources and methods are provided in the Technical Appendix.

Using microbiological criteria from current guidelines (*5*), we defined 2 mutually exclusive groups. One positive sputum sample defined NTM-PI, whereas >1 positive sputum sample for the same species or 1 positive bronchoscopic or biopsy specimen defined NTM-PD. We disregarded *Mycobacterium gordonae* isolates and excluded persons with prior (1998–2000) NTM isolation. We calculated 12 propensity scores (estimating the patient-level likelihood of species–condition combinations), 1 for each species–condition combination of interest, comprising the species groups *M. avium* complex (MAC), *M. xenopi*, *M. fortuitum*, M. *abscessus*, *M. kansasii*, and other, according to the condition-states NTM-PI and NTM-PD. We sought to match each patient with NTM (exposed person) to an Ontario resident without NTM (unexposed control) who shared the age (years), sex, and index date (± 90 days), and had a propensity score value within 0.2 × SD of the exposed patient (*15*). Index date was date of first positive culture for exposed patients and was randomly assigned to potential unexposed controls by using a random number generator.

We characterized our cohort by demographics, underlying conditions, and healthcare utilization. Primary analyses compared survival of propensity score–matched exposed with unexposed persons, for each species–condition group (e.g., patients with MAC NTM-PD vs. their population-matched unexposed controls). We also compared, for individual NTM species-groups and all species-groups combined, survival of patients with any isolation (NTM-PI or NTM-PD) to their propensity score–matched controls and survival of all patients with NTM-PD or NTM-PI (without matching). We also calculated standardized mortality ratios, using age- (in 5-year strata) and sex-specific all-cause death rates in the Ontario general population during the study period (2001–2013), as an intuitive mortality risk assessment without full adjustment. To study whether NTM-PD with >1 NTM species (multispecies NTM-PD) affected survival, we compared the survival of NTM-PD patients infected with 1 species with survival of patients who fulfilled criteria for >1 species. Finally, we examined associations between demographic and clinical factors with death.

Follow-up began on the index date and ended at death or end of the study period, whichever came first. Survival analyses comprised Cox proportional hazards models. Including low-frequency covariates (Table 1) in propensity score calculation led to substantially fewer NTM patients successfully matching to an unexposed person. These covariates were explored for inclusion by using a Hosmer-Lemeshow approach (inclusion if >10% effect on point estimate [*18*]); none were retained. Comparisons between groups that were not propensity-matched (NTM-PD vs. NTM-PI and single-species vs. multispecies NTM-PD) included all Ontario residents with NTM species–conditions of interest and were adjusted for age, sex, and covariates used to characterize the cohort in our study. For survival analyses of single-species versus multispecies NTM-PD, we addressed immortal time bias (time to second species infection inflating survival of patients with multispecies NTM-PD) using status of single-species versus multispecies NTM as a time-varying covariate. Secondary survival analyses excluded patients who died within 30 days after NTM index date, assuming that early death was unrelated to NTM.

**Table 1:** Characteristics of patients with NTM pulmonary disease and matched persons without NTM for MAC, *Mycobacterium xenopi*, and *M. abscessus*, Ontario, Canada, 2001–2013*

Characteristic	MAC				*M. xenopi*				*M. abscessus*		
Disease, n = 5,543	Control, n = 5,543	SDM	Disease, n = 1,975	Control, n = 1,975	SDM	Disease, n = 201	Control, n = 201	SDM
Female sex, %	53	53	0		45	45	0		58	58	0
Median age, y (IQR)	70 (58–78)	70 (58–78)	0		70 (58–78)	70 (58–78)	0		64 (47–74)	64 (47–74)	0
Underlying condition, %											
Asthma	31	29	0.06		36	31	0.1		25	31	0.13
COPD	45	52	0.14		51	56	0.11		32	34	0.04
Diabetes	19	25	0.15		21	24	0.09		12	15	0.1
Rheumatoid arthritis	3	3	0.01		3	3	0.01		<3†	<3†	0.13
Chronic kidney disease	7	9	0.05		8	8	0		5	5	0
GERD	16	19	0.05		17	18	0.02		11	13	0.05
Bronchiectasis	10	5	0.17		7	5	0.06		9	8	0.04
Interstitial lung disease	5	4	0.06		6	4	0.11		6	4	0.1
Lung cancer	5	3	0.09		7	4	0.13		4	5	0.07
HIV infection‡	2	0.2	0.18		2	0	0.18		0	0	
Solid organ transplant‡	0.5	0.1	0.07		1	<0.3†	0.13		3	0	0.25
BMT‡	0.4	<0.1	0.06		1	<0.3†	0.12		<3†	0	0.14
Cystic fibrosis‡	0.5	<0.1	0.07		0.4	<0.3†	0.04		8	0	0.42
Prior tuberculosis‡	2	0	0.18		2	<0.3†	0.19		4	0	0.29
Hospitalizations§	0.31 ± 0.76	0.32 ± 0.77	0.01		0.39 ± 0.84	0.37 ± 0.84	0.02		0.29 ± 0.68	0.30 ± 0.71	0.02
ED visits§	0.85 ± 1.15	0.80 ± 1.56	0.03		0.88 ± 1.20	0.80 ± 1.51	0.06		0.55 ± 0.88	0.69 ± 1.37	0.12
ACG diagnoses	9.7 ± 3.8	9.5 ± 3.8	0.05		10.1 ± 4.0	9.8 ± 3.9	0.06		8.6 ± 3.9	8.5 ± 4.2	0.03

*Matched according to age (years), sex, index date (± 90 d), and propensity score (estimating the patient-level likelihood of species-specific NTM pulmonary disease) value within 0.2 × SD of the exposed patient. NTM pulmonary disease was defined as the presence of >1 positive sputum sample for the same species or 1 positive bronchoscopic or biopsy specimen. Controls were persons without NTM matched by age, sex, index date, and propensity score. ACG; adjusted clinical group diagnoses using the ACG case mix system (*16*); BMT, hematopoietic stem cell transplant; COPD, chronic obstructive pulmonary disease; ED; emergency department; GERD, gastresophageal reflux disease; IQR, interquartile range; MAC, *Mycobacterium avium* complex; NTM, nontuberculous mycobacteria; SDM; standardized difference of the mean (value of <0.1 generally considered not significant) (*17*). Empty cells indicate value undefined. †Range reported because of small cell size (direct or by inference), which in accordance with privacy regulations cannot be reported. ‡Baseline characteristics not included in the propensity score because of their effect to substantially reduce successful matching of exposed cases with unexposed controls. Inclusion of these variables as covariates was explored, but none significantly altered the hazard ratio point estimates. §Number of events in year before index date.

## Results

During the 13-year study period, 20,617 Ontario residents had incident NTM isolation from respiratory tract specimens: 10,936 (53%) with NTM-PI and 9,681 (47%) with NTM-PD. Propensity score matching was successful for 9,967 (91%) NTM-PI patients and 8,469 (87%) NTM-PD patients. Compared with matched patients, patients who could not be matched to unexposed controls were older (NTM-PI, median 74 vs. 64 years, p<0.001; NTM-PD, median 72 vs. 70 years, p<0.001) and had higher frequencies of underlying conditions with higher mean adjusted clinical group case mix (*16*) numbers (NTM-PI 12.6 vs. 8.9, p<0.001; NTM-PD 12.4 vs. 9.8, p<0.001) (Technical Appendix Table 1). In addition, unmatched patients had substantially lower survival than did matched patients at 1 year (76.1% vs. 91.0%) and 5 years (46.3% vs. 76.0%) for all NTM-PI and at 1 year (75.3% vs. 85.7%) and 5 years (47.7% vs. 65.4%) for all NTM-PD.

We observed small differences in sex distribution by species, whereby NTM-PD with MAC and *M. abscessus* was seen more commonly in female patients and the other NTM species were seen more commonly in male patients (Table 1, 2; Technical Appendix Tables 2–4). Combining all species, the sexes were similarly represented for NTM-PD and NTM-PI. The median (interquartile range) age ranged from 60 (43–74) to 70 (58–78) years for the different species–condition groups and for combining species was 65 (49–77) years for NTM-PI and 70 (58–78) years for NTM-PD. Patients had a high prevalence of underlying conditions by adjusted clinical group numbers (7.3–10.1), including asthma (25%–36%), chronic obstructive pulmonary disease (COPD; 25%–52%), diabetes (12%–24%), chronic kidney disease (3%–8%), and gastresophageal reflux disease (11%–20%), ranging by species–condition groups. Covariates were generally balanced between the matched groups.

**Table 2:** Characteristics of patients with NTM pulmonary isolation and matched persons without NTM for MAC, *Mycobacterium xenopi*, and *M. abscessus*, Ontario, Canada, 2001–2013*

Characteristic	MAC				*M. xenopi*				*M. abscessus*		
Isolation, n = 5,242	Control, n = 5,242	SDM	Isolation, n = 2,693	Control, n = 2,693	SDM	Isolation, n = 162	Control, n = 162	SDM
Female sex, %	51	51	0		48	47	0		49	49	0
Median age, y (IQR)	65 (49–76)	65 (49–76)	0		65 (48–76)	65 (48–76)	0		60 (43–74)	60 (43–74)	0
Underlying condition, %											
Asthma	29	25	0.09		31	26	0.1		25	20	0.12
COPD	36	41	0.1		38	42	0.09		25	26	0.01
Diabetes	18	23	0.12		19	21	0.06		15	16	0.03
Rheumatoid arthritis	3	2	0.06		3	3	0.01		<4†	5	0.09
Chronic kidney disease	6	7	0.05		7	8	0.03		5	6	0.05
GERD	14	17	0.08		15	17	0.05		14	13	0.02
Bronchiectasis	7	4	0.13		6	4	0.09		6	5	0.03
Interstitial lung disease	3	2	0.06		3	3	0.03		<4†	4	0.11
Lung cancer	2	2	0.02		2	2	0.04		<4†	<4†	0.18
HIV infection‡	1	0.2	0.13		2	<0.2†	0.19		0	0	
Solid organ transplant‡	0.3	0.3	0		0.5	<0.2†	0.07		0	<4†	0.11
BMT‡	0.1	0.1	0		<0.2†	<0.2%†	0.04		0	0	
Cystic fibrosis‡	0.4	0.1	0.05		0.2	0	0.07		5	<4†	0.27
Prior tuberculosis‡	3	<0.1†	0.23		3	<0.2%†	0.25		<4†	0	0.19
Hospitalizations§	0.31 ± 0.77	0.30 ± 0.76	0.01		0.33 ± 0.78	0.31 ± 0.78	0.02		0.19 ± 0.53	0.23 ± 0.60	0.09
ED visits§	0.83 ± 1.22	0.79 ± 1.79	0.02		0.81 ± 1.21	0.77 ± 1.59	0.03		0.49 ± 0.88	0.46 ± 1.08	0.03
ACG diagnoses	9.0 ± 4.1	8.7 ± 4.1	0.06		9.1 ± 4.2	8.8 ± 4.2	0.08		7.3 ± 4.3	7.4 ± 4.4	0.01

*Matched according to age (years), sex, index date (± 90 d), and propensity score (estimating the patient-level likelihood of species-specific NTM pulmonary isolation) value within 0.2 × SD of the exposed patient. NTM pulmonary isolation was defined as the presence of 1 positive sputum specimen. Controls were persons without NTM matched by age, sex, index date, and propensity score. ACG, adjusted clinical group diagnoses using the ACG case mix system (*16*); BMT, hematopoietic stem cell transplant; COPD, chronic obstructive pulmonary disease; ED, emergency department; GERD, gastresophageal reflux disease; IQR, interquartile range; MAC, *Mycobacterium avium* complex; NTM, nontuberculous mycobacteria; SDM, standardized difference of the mean (value <0.1 generally considered not significant) (*17*). Blank cells indicate value undefined. †Range reported because of small cell size (direct or by inference), which according to privacy regulations cannot be reported. ‡Baseline characteristics not included in the propensity score because of their effect to substantially reduce successful matching of exposed cases with unexposed controls. Inclusion of these variables as covariates was explored, but none significantly altered the hazard ratio point estimates. §Number of events in year before index date.

**Table 4:** Survival estimates for patients with incident pulmonary NTM disease and with NTM isolation, Ontario, Canada, 2001–2013*

Species, group	Total	1-y survival, %	5-y survival, %	SMR† (95% CI)	Crude HR (95% CI)	Adjusted‡ HR (95% CI)
All						
Disease	9,681	84.4	63.1	2.83 (2.74–2.92)	1.49 (1.42–1.56)	1.23 (1.17–1.28)
Isolation	10,936	89.7	73.4	2.30 (2.22–2.38)	1.00 (ref)	1.00 (ref)
MAC						
Disease	6,323	85.7	64.7	2.59 (2.49–2.69)	1.40 (1.32–1.49)	1.16 (1.09–1.24)
Isolation	5,756	89.5	73.2	2.27 (2.16–2.38)	1.00 (ref)	1.00 (ref)
*M. xenopi*						
Disease	2,263	80.2	56.8	3.49 (3.29–3.70)	1.71 (1.57–1.86)	1.39 (1.27–1.52)
Isolation	2,932	88.7	71.9	2.39 (2.23–2.55)	1.00 (ref)	1.00 (ref)
*M. fortuitum*						
Disease	265	84.9	64.8	2.70 (2.21–3.18)	1.47 (1.17–1.85)	1.17 (0.92–1.48)
Isolation	714	89.8	76.0	2.63 (2.27–2.99)	1.00 (ref)	1.00 (ref)
*M. abscessus*						
Disease	245	88.6	72.8	2.23 (1.71–2.74)	1.25 (0.87–1.80)	1.14 (0.76–1.72)
Isolation	185	91.4	77.1	2.10 (1.46–2.74)	1.00 (ref)	1.00 (ref)
*M. kansasii*						
Disease	158	84.2	62.3	4.37 (3.37–5.37)	0.91 (0.65–1.29)	1.15 (0.78–1.68)
Isolation	106	82.1	52.0	3.97 (2.89–5.05)	1.00 (ref)	1.00 (ref)
Other						
Disease	427	84.8	66.7	3.08 (2.62–3.55)	1.55 (1.29–1.87)	1.27 (1.04–1.55)
Isolation	1,243	92.8	77.6	1.94 (1.72–2.15)	1.00 (ref)	1.00 (ref)

*Comparisons between all registered Ontario residents with incident species-specific NTM isolation and NTM disease respectively; Matching not required for this analysis, and so adjustment was made for all covariates of interest. NTM disease was defined as >1 positive sputum for the same species or 1 positive bronchoscopic or biopsy specimen. NTM isolation was defined as 1 positive sputum sample for NTM. MAC, mycobacterium avium complex; NTM, nontuberculous mycobacterium; ref, reference; SMR, standardized mortality ratio. †Standardized by sex and age in 5-y strata using data for the Ontario population. ‡Adjusted for sex, age, income quintile, location, Adjusted Clinical Group case mix system, baseline underlying conditions (asthma, chronic obstructive pulmonary disease, diabetes, HIV infection, rheumatoid arthritis, chronic kidney disease, gastresophageal reflux disease, bronchiectasis, interstitial lung disease, cystic fibrosis, prior tuberculosis, lung cancer, solid organ transplantation or bone marrow transplantation), health use (number of hospitalizations and emergency department visits in year before index date), and NTM disease diagnosis during follow-up as time-varying covariate (for NTM isolation group only).

Kaplan-Meier plots for any NTM (NTM-PD or NTM-PI), including both matched and unmatched patients, by species group, revealed apparently distinct death rates; survival was highest for patients with *M. abscessus* and lowest for patients with *M. kansasii* (Figure). In this crude survival comparison, uncontrolled for age, sex, or any other variables, we found a statistically significant difference among curves (p<0.001, log-rank) and significant differences in most pairwise comparisons between species.

**Figure:**
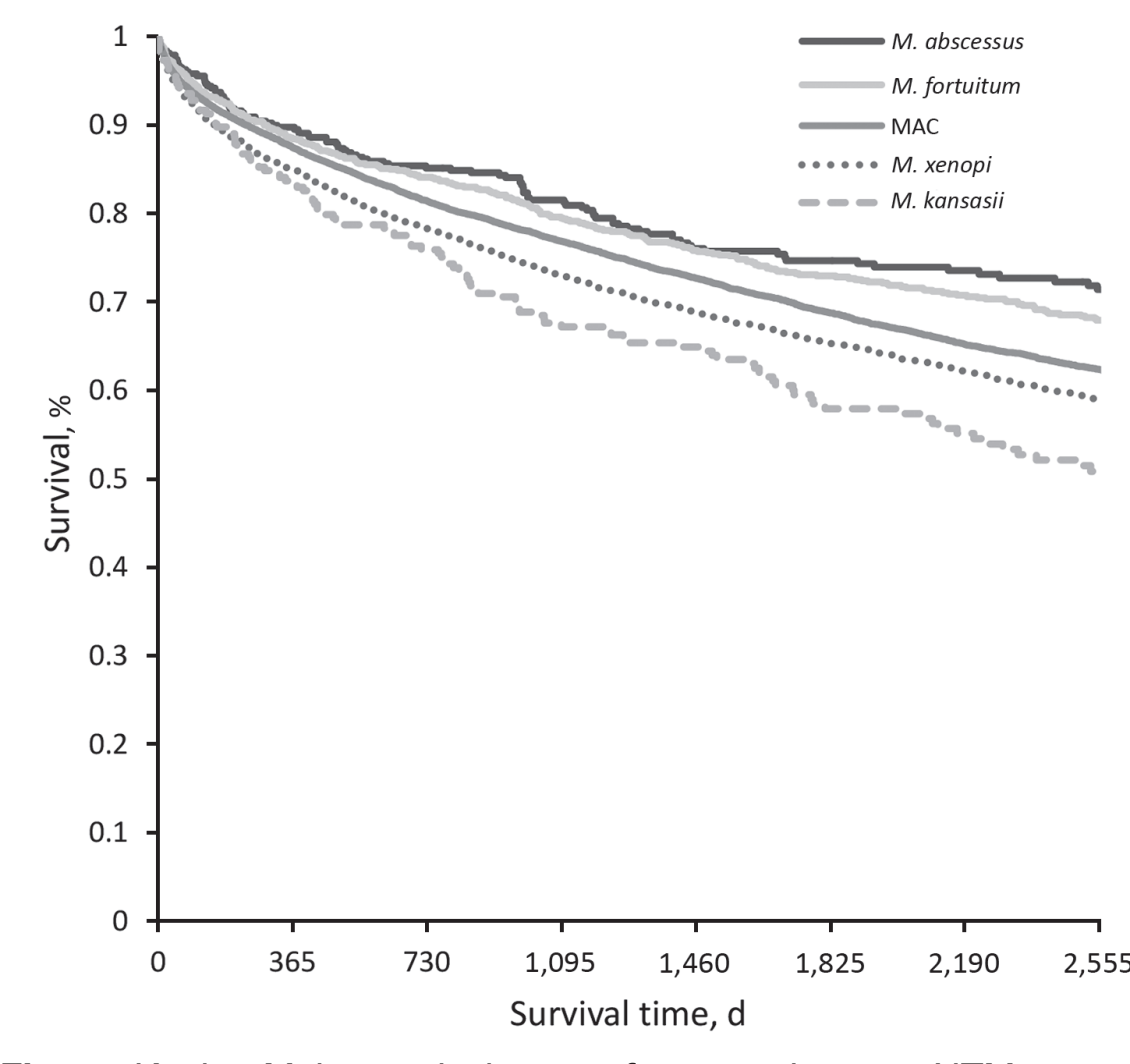
Kaplan-Meier survival curves for any pulmonary NTM isolation, by species group, Ontario, Canada, 2001–2013. Curve comprises all matched and unmatched patients identified during the study period. There is a statistically significant difference among curves (p<0.001, log-rank) in crude survival comparison, uncontrolled for any other variables. Differences between individual species pairs statistically significant (p<0.00005) for all pairs except *Mycobacterium abscessus* versus *M. fortuitum* (p = 0.19), *M. abscessus* versus *Mycobacterium avium* complex (p = 0.14), and *M. fortuitum* versus *Mycobacterium avium* complex (MAC) (p = 0.50).

Compared with age-, sex-, and propensity-matched unexposed controls, 1- and 5-year survivals were numerically lower for patients with NTM in all groups, regardless of species or condition (NTM-PI or NTM-PD) (Table 3). Hazard ratios (HRs) for death were elevated for all species–condition groups, but those for *M. abscessus* isolation (HR 1.39, 95% CI 0.94–2.07), and *M. fortuitum* disease (HR 1.25, 95% CI 0.96–1.63) were not statistically significant.

**Table 3:** Survival estimates for patients with pulmonary NTM and for matched controls, Ontario, Canada, 2001–2013*

Group	Any NTM (isolation or disease)				NTM isolation only				NTM disease		
	Total	5-y survival, %	HR (95% CI)		Total	5-y survival, %	HR (95% CI)		Total	5-y survival, %	HR (95% CI)
NTM	18,436	71.3	1.47 (1.42–1.51)		9,967	76.1	1.33 (1.27–1.39)		8,469	65.6	1.63 (1.56–1.70)
Control	18,436	80.8	1.00 (ref)		9,967	82.6	1.00 (ref)		8,469	78.7	1.00 (ref)
MAC	10,785	71.2	1.45 (1.39–1.51)		5,242	75.9	1.33 (1.25–1.41)		5,543	66.7	1.57 (1.48–1.66)
Control	10,785	80.4	1.00 (ref)		5,242	82.3	1.00 (ref)		5,543	78.5	1.00 (ref)
*M. xenopi*	4,668	68.3	1.54 (1.45–1.64)		2,693	74.5	1.32 (1.22–1.44)		1,975	59.9%	1.84 (1.69–2.01)
Control	4,668	79.9	1.00 (ref)		2,693	81.5	1.00 (ref)		1,975	77.7%	1.00 (ref)
*M. fortuitum*	890	76.4%	1.39 (1.21–1.60)		654	78.8	1.47 (1.24–1.73)		236	69.7	1.25 (0.96–1.63)
Control	890	84.9%	1.00 (ref)		654	86.4	1.00 (ref)		236	80.7	1.00 (ref)
*M. abscessus*	363	80.5	1.45 (1.09–1.92)		162	82.2	1.39 (0.94–2.07)		201	79.2	1.49 (1.00–2.21)
Control	363	86.6	1.00 (ref)		162	85.7	1.00 (ref)		201	87.3	1.00 (ref)
*M. kansasii*	236	60.2	2.29 (1.76–2.97)		92	55.3%	2.02 (1.37–3.00)		144	63.5	2.53 (1.78–3.58)
Control	236	84.0	1.00 (ref)		92	85.3%	1.00 (ref)		144	83.0	1.00 (ref)
Other species	1,494	77.7	1.28 (1.13–1.43)		1124	80.0%	1.20 (1.04–1.38)		370	70.7	1.51 (1.22–1.88)
Control	1,494	82.4	1.00 (ref)		1124	83.2%	1.00 (ref)		370	79.8	1.00 (ref)

*Matched by age (years), sex, index date (± 90 d), and propensity score (estimating the patient-level likelihood of species–condition combinations) value within 0.2 × SD of the exposed patient. NTM isolation was defined as 1 positive sputum specimen for NTM. NTM disease was defined as >1 positive sputum for the same species or 1 positive bronchoscopic or biopsy specimen. Controls were persons without NTM matched by age, sex, index date, and propensity score. HR, hazard ratio; MAC, *Mycobacterium avium* complex; NTM, nontuberculous mycobacterium; ref, referent.

As illustrated by the standardized mortality ratios, adjusted for sex and 5-year age stratum, death rates were increased above expected for the Ontario general population for all groups compared (Table 4). In adjusted comparisons, patients with NTM-PD (vs. NTM-PI) had higher death rates for all species combined, as well as for MAC and *M. xenopi* individually (Table 4). For the other species groups, although the HRs were similar in magnitude to that for MAC, death rates were not significantly higher. Compared with the 9,061 patients who had single-species NTM-PD, the 620 patients with multispecies NTM-PD (any combination of NTM species) had higher rates of death (HR 1.19, 95% CI 1.04–1.34). Death rates also were higher for the subgroup of MAC plus *M. xenopi* (n = 354) versus either species alone (n = 8,059) (HR 1.23, 95% CI 1.04–1.45). The sample size was inadequate to assess other species combinations. In the multivariable analysis of baseline factors for association with death among all patients with incident NTM-PD (matched and unmatched patients), increasing age, male sex, low income, and underlying conditions were all associated with reduced survival (Table 5). Compared with MAC (reference species group), *M. xenopi* was the only species associated with a significantly higher death rate. The results of secondary survival analyses that excluded patients who died within 30 days did not differ significantly from any primary survival analyses.

**Table 5:** Multivariable associations between baseline clinical variables and death among all patients with incident NTM pulmonary disease, Ontario, Canada, 2001–2013*

Variable	Value, n = 9,681	Adjusted† HR (95% CI)	p value
Male sex, no. (%)	49.1	1.47 (1.38–1.57)	<0.0001
Median age, y (IQR)	70 (58–78)	1.05 (1.05–1.05)	<0.0001
Income quintile,‡ %			
1 (lowest; reference)	26.7	–	–
2	21.7	0.93 (0.86–1.02)	0.1267
3	18.0	0.90 (0.82–0.99)	0.0332
4	16.3	0.86 (0.78–0.95)	0.0024
5	17.1	0.82 (0.74–0.90)	<0.0001
Residential setting,§ %			
Urban (reference)	89.5	–	–
Suburban	7.8	0.98 (0.87–1.10)	0.7091
Rural	2.7	1.04 (0.87–1.25)	0.6783
ACG number,¶ %			
0–5 (reference)	11.9	–	–
6–10	42.5	1.21 (1.05–1.39)	0.0084
>11	45.6	1.44 (1.25–1.66)	<0.0001
Underlying condition, %			
Asthma	35.1	0.88 (0.82–0.94)	0.0003
COPD	51.3	1.38 (1.29–1.48)	<0.0001
Diabetes	19.9	1.05 (0.97–1.13)	0.2505
Rheumatoid arthritis	3.5	1.19 (1.01–1.39)	0.0339
Chronic kidney disease	8.1	1.40 (1.27–1.55)	<0.0001
GERD	17.3	0.93 (0.86–1.01)	0.0832
Bronchiectasis	14.2	0.76 (0.69–0.84)	<0.0001
Interstitial lung disease	8.1	1.51 (1.37–1.68)	<0.0001
Lung cancer	8.0	3.03 (2.78–3.32)	<0.0001
HIV infection	1.8	3.56 (2.81–4.49)	<0.0001
Cystic fibrosis	1.0	1.95 (1.37–2.77)	0.0002
Solid organ transplant	1.4	1.06 (0.81–1.38)	0.6849
Bone marrow transplant	0.6	2.77 (1.93–3.97)	<0.0001
Prior tuberculosis	1.8	0.66 (0.50–0.87)	0.0037
Hospitalizations,# mean ± SD	0.41 ± 0.93	1.09 (1.05–1.14)	<0.0001
Emergency department visits,# mean ± SD	0.93 ± 1.24	1.16 (1.13–1.20)	<0.0001
NTM species, %			
MAC (reference)	65.3	–	–
* M. xenopi*	23.4	1.22 (1.13–1.31)	<0.0001
* M. fortuitum*	2.7	1.02 (0.84–1.23)	0.8538
* M. abscessus*	2.5	0.98 (0.78–1.24)	0.8841
* M. kansasii*	1.6	1.25 (0.99–1.57)	0.0636
All other species	4.4	0.94 (0.80–1.10)	0.4306

*Multivariable Cox proportional hazards model including all 9,681 registered Ontario residents with incident NTM pulmonary disease (>1 positive sputum sample for the same species or 1 positive bronchoscopic or biopsy specimen). No matching required for this analysis, and so all covariates of interest were assessed. ACG, Adjusted Clinical Group; COPD, chronic obstructive pulmonary disease; GERD, gastresophageal reflux disease; IQR, interquartile range; MAC, mycobacterium avium complex; NTM, nontuberculous mycobacteria. Dashes indicate reference level for the variable (values not calculated). †Adjusted for sex, age, income quintile, location, ACG case mix system, baseline underlying conditions (asthma, COPD, diabetes, HIV, rheumatoid arthritis, chronic kidney disease, GERD, bronchiectasis, interstitial lung disease, cystic fibrosis, prior tuberculosis, lung cancer, solid organ transplantation or bone marrow transplantation), health use (number of hospitalizations and emergency department visits in year before index date), and NTM disease diagnosis during follow-up as time-varying covariate (for NTM isolation group only). ‡Totals do not add to 100% because of missing income data in 0.4% of patients §Residential setting characterized by Rural Index of Ontario (*19*). ¶Number of ACG diagnoses using the ACG case mix system (*16*). #Number of events in the year before entry.

## Discussion

Our population-based cohort study matched >18,000 patients who had microbiologically confirmed pulmonary NTM to unexposed controls and clearly demonstrates an increased risk for death with both NTM-PD and NTM-PI. The veracity of this prognostic information is supported by the rigorous survival comparison with unexposed persons matched by age, sex, and the propensity to have NTM. The relatively small sample sizes might account for the fact that we did not observe a significant risk for death in some individual subgroups (NTM-PI with *M. abscessus* and NTM-PD with *M. fortuitum*) compared with unexposed controls.

Previous studies demonstrated high rates of death with NTM (*7*–*11*), but none compared with matched population-based controls. In addition, most prior studies comprised patients from NTM clinics, which permitted careful clinical characterization but most likely led to substantial bias in patient selection (*7*,*8*,*10*). Although our 5-year mortality estimates (26.6% for NTM-PI and 36.9% for NTM-PD) were of generally similar magnitude to prior studies (*7*–*11*), parsing those studies by cohort type reveals that those comprising single NTM clinics tended to report lower rates of death (*7*,*8*,*10*). In the single-clinic study from the United States, which had the lowest 5-year death rate of 18% (*10*), the median age at diagnosis was 55 years, compared with 70 years in our study, which probably explains much of the difference in death. The 2 single-clinic studies from Japan, comprising exclusively patients with MAC pulmonary disease (MAC-PD), reported 5-year death rates of 23.1% and 23.9% (*7*), compared with 33.5% for MAC-PD patients in our study, despite similar ages among the 3 studies. In 1 of the studies, treated MAC-PD patients had a 5-year risk for death of 22.2%, whereas untreated chronic MAC-PD patients had a 5-year risk for 33.3% (*8*), similar to the patients in our study. Perhaps most MAC-PD patients in Ontario have untreated chronic disease, whereas patients in NTM clinics are more likely to be treated, which might reduce their risk for death.

A comprehensive population-based study from Denmark observed death rates of 39.7% for MAC-PD and 51.0% for *M. xenopi* disease, compared with 36.3% and 43.2% in our study. The higher rates of death in the Denmark study are interesting considering that the cohort was younger and had a smaller proportion of patients with COPD, diabetes, and renal disease. However, because the Denmark study did not provide detailed clinical characteristics and comprised approximately one third *M. gordonae* patients (*11*), comparing that cohort with the cohort in our study is difficult. Despite methodologic differences, the odds ratio (OR) for death in a Medicare-based study in the United States (OR 1.4, 95% CI 1.3–1.6) (*3*) was similar to our HR for death in survival analysis (HR 1.63, 95% CI 1.56–1.70). The US study comprised only patients who were >65 years, not enrolled in a health maintenance organization, and identified with NTM infection using codes from the International Classification of Diseases, Ninth Revision (ICD-9). Restricting our cohort to patients >65 years of age would exclude >37% of all patients with NTM-PD. Although the use of ICD-9 codes to identify patients with NTM infection has not been validated, this method appears to be specific but relatively insensitive (*20*), introducing bias depending on characteristics of NTM-infected patients not detected by this method.

The risk for death we observed with NTM-PD was generally greater than with NTM-PI, which was significant for all species combined, MAC, and *M. xenopi*. The lack of a significant difference in survival between NTM-PD and NTM-PI for the other species might have reflected the relatively small number of cases. Our results are consistent with findings in the population-based study from Denmark (*11*). In the Denmark study, 709 patients with 1 positive specimen tended to survive longer than the 238 patients with 2 or 3 positive specimens, who in turn tended to survive longer than the 335 patients who had >3 positive specimens (p = 0.07). The difference in statistical significance probably is due to sample size because our study had far more patients.

The significantly higher death rate for patients who had even 1 positive sputum specimen for MAC or *M. xenopi* is of particular interest. In some cases a positive sputum sample could be insignificant, representing contamination or transient presence of the organism, which would presumably not be associated with increased risk for death. In other cases, a positive sputum sample might represent the 1-time identification of a chronically present organism, which might or might not be causing significant disease. In this latter group, the presence of only 1 positive sample could be explained either by inadequate sampling or difficulty in identifying a true pathogen present in low numbers. Either way, some patients designated as having NTM-PI probably had true disease. The lack of data about negative cultures precludes further exploration of this issue.

As with others’ findings, we identified variable survival among patients with different NTM species. Our unadjusted analyses are consistent with the Denmark study; patients with *M. abscessus* and *M. fortuitum* had lower death rates than patients with MAC, who in turn had lower death rates than patients with *M. xenopi* (*11*). However, in our adjusted analysis, survival with *M. abscessus* and *M. fortuitum* pulmonary disease did not differ from survival with MAC disease. The worse crude survival observed with *M. xenopi* and *M. kansasii* persisted in adjusted analyses only for *M. xenopi*. The reason for the worse prognosis with *M. xenopi*, despite adjustment for age, sex, and underlying conditions, is not clear. There could be residual confounding related to cavitation and COPD, both commonly present with *M. xenopi* disease. Patients with *M. xenopi* disease have far higher rates of cavitation than do patients with MAC (46% vs. 16%; p = 0.01) (*21*), and cavitation is consistently associated with death (*7*,*8*,*10*). *M. xenopi* is also associated with COPD (*11*), and although both studies adjusted for COPD, the severity of COPD could be greater among patients with *M. xenopi* infection. Inadequately measured covariates could potentially explain the poor survival among patients infected with *M. xenopi*. Alternatively, perhaps *M. xenopi* disease is a more lethal condition. The lower survival observed with *M. kansasii* (compared with MAC) in unadjusted analysis, which did not persist in adjusted analysis, is most likely explained largely by the high proportion of male patients, COPD, and HIV infection among *M. kansasii* patients. Regardless, the adjusted analysis did not find that *M. kansasii* was associated with higher death rates than MAC, perhaps as expected because the former is believed to be the most readily curable of the NTM pulmonary pathogens (*5*), despite a high prevalence of cavitary disease (*22*).

The analysis of risk factors for death among patients with NTM-PD provided results generally consistent with those of previous studies. As in our study, increasing age (*7*,*11*), male sex (*3*,*7*,*11*), and underlying conditions (*3*,*11*) are repeatedly identified as risk factors for death. Other factors, including COPD, asthma, bronchiectasis, and other diseases, have been less studied. Our finding that COPD is associated with death (HR 1.38, 95% CI 1.29–1.48) seems plausible, not only because of the death intrinsic to COPD, but also because fibrocavitary NTM-PD develops more often in patients with COPD (*5*), and cavitation is associated with lower survival (*7*,*8*,*10*). Our finding that asthma is associated with a lower risk for death (HR 0.89, 95% CI 0.83–0.95) is perhaps surprising; it might be confounded by an association between asthma and nodular bronchiectatic NTM-PD, which has a better prognosis than cavitary disease (*7*,*8*,*10*). Although 1 previous study reported that NTM-PD patients with asthma had a higher risk for death (OR 1.7, 95% CI 1.1–2.7) (*3*), NTM-PD was identified by ICD-9 coding, which might overlook a large proportion of NTM-PD and thus introduce bias. The presence of bronchiectasis in NTM-PD presumably makes cavitation less likely to be present, and so our finding of lower death rates in the presence of bronchiectasis (HR 0.77, 95% CI 0.70–0.84) seems plausible and is consistent with a prior report (*3*). The significant associations that we observed between death and interstitial lung disease, lung cancer, HIV infection, cystic fibrosis, and bone marrow transplantation all seem plausible given the risks for death generally conferred by these clinical factors. Some underlying conditions undoubtedly emerged after the index date in affected patients and in unexposed controls. We elected to ignore any mortality effect of subsequently emerging underlying conditions, favoring the development of a mortality estimate based on information at the time of diagnosis.

Given the high frequency of bronchiectasis in NTM-PD (*5*), it is noteworthy that bronchiectasis in our study was uncommon, measured as 8.5% and 6.1% among matched NTM-PD and NTM-PI patients, respectively, and 14.2% and 10.5% among all NTM-PD and NTM-PI patients. Our reliance on a simple unvalidated diagnostic code definition probably failed to identify the presence of bronchiectasis in many patients. The underappreciation of bronchiectasis might have been greater among the groups of patients with NTM, wherein one would expect a high proportion of bronchiectasis. Accordingly, assuming that bronchiectasis per se increases death, our death estimates in NTM groups might be overestimates.

Our work has several important limitations. First, because of an absence of clinical data, our definition of NTM-PD is based exclusively on microbiology, which probably misclassifies some patients with NTM colonization as having disease. Nevertheless, this misclassification rate most likely is small because microbiological-based definitions of NTM-PD exhibit high accuracy (*11*,*20*,*23*,*24*). Furthermore, the resulting effect of diagnostic misclassification would probably not significantly alter our findings. Misclassifying some patients with NTM-PI as NTM-PD would be expected to incorrectly place some patients with milder illness into the NTM-PD group and result in an attenuated apparent death attributable to NTM-PD. Therefore, the NTM-PD death rate is perhaps somewhat greater than we observed. There could be a stage migration phenomenon (*25*), by selective misclassification of the “more severe” NTM-PI as NTM-PD, apparent death could be reduced in both NTM-PI and NTM-PD. The same effect is also at play in our definition of NTM-PD with respect to the time between a first and a second positive sputum culture. Rather than arbitrarily defining a time period between a first and a second positive sputum culture, which would separate patients with “initial NTM-PI progressing to NTM-PD” and “initial NTM-PD,” we defined all patients with 2 positive sputa at any time during the study period as having NTM-PD. The index date was always the date of the first positive sputum, which introduces an immortal time bias in the patients with NTM-PDd, defined as the time between the first and second positive sputum sample and leads to some underestimation of the associated mortality of NTM. Second, the lack of comprehensive medication information precludes assessing the effect of antimycobacterial treatment on survival and limits our ability to control for severity of some comorbid illnesses, such as COPD. Third, we were unable to ascertain cause of death for patients in our study. Prior studies have yielded mixed results in this area. Two studies of MAC-PD from separate NTM clinics in Japan reported that most patients died of causes other than their NTM-PD (*7*,*8*), whereas a study from 1 NTM clinic in the United States found that most deaths were NTM-related (*10*). We suspect that the higher proportion of deaths from NTM in the US study occurred because the patients were substantially younger at diagnosis, making deaths from other causes much less likely. Fourth, our propensity score matching was unsuccessful for 9% of NTM-PI patients and 13% of NTM-PD patients, and unmatched patients were older and had more underlying conditions. Although the effect of omitting these patients on our calculated HRs is not clear, an underestimation of the true risk for death is likely, in that unmatched NTM patients had substantially lower survival than did matched NTM patients. Fifth, the lack of data on patients with negative mycobacterial cultures precluded using such patients as unexposed controls. Other factors detected by clinicians and triggering a request to collect respiratory specimens for mycobacterial studies might have confounded our mortality estimates.

In summary, patients with NTM-PD have significantly lower survival than do appropriately matched population controls. This increasingly common health problem is clearly associated with not only substantial illness but with death as well. Further work should clarify the mortality effects of co-existing conditions, such as COPD, asthma, and bronchiectasis, infection with different NTM species, especially *M. xenopi*, specific antimicrobial treatment of the NTM infection, and cause of death among NTM patients.

Technical Appendix Data sources and definitions, statistical analysis, and additional results.
