# Supplementary material for: Pulmonary Nontuberculous Mycobacteria–Associated Deaths, Ontario, Canada, 2001–2013
Source: Emerg Infect Dis. 2017 Mar;23(3):468–76. doi: 10.3201/eid2303.161927 (PMC5382758; doi:10.3201/eid2303.161927)
Supplement: Technical Appendix — Data sources and definitions, statistical analysis, and additional results. [file 16-1927-Techapp-s1.pdf]

Article DOI: http://dx.doi.org/10.3201/eid2303.161927

Pulmonary Nontuberculous Mycobacteria–Associated Deaths, Ontario, Canada, 2001–2013

Technical Appendix

Data Sources and Definitions

We considered all persons in Ontario's Registered Persons Database during January 1, 2001–December 31, 2013. This database, which contains demographic and vital status information about every Ontario resident with a valid health card, was used to identify and characterize our cohort. Ontario residents have universal public health insurance under the Ontario Health Insurance Plan (OHIP), the single payer for medically necessary services. We excluded persons who were ineligible for OHIP coverage (e.g., immigrants during their initial 3 months of residence). By using linked population-based laboratory data from the Tuberculosis and Mycobacteriology section of Public Health Ontario, we identified the index date of pulmonary nontuberculous mycobacterium (NTM) infection. Using microbiological criteria from current guidelines (*1*), we defined 2 mutually exclusive groups. Patients with 1 positive sputum sample were classified as having NTM pulmonary isolation (NTM-PI), and patients with >1 positive sputum sample for the same species or 1 positive bronchoscopic or biopsy specimen were classified as having NTM pulmonary disease (NTM-PD). We disregarded *Mycobacterium gordonae* isolates because this species is almost uniformly a contaminant (*1*), and we excluded persons with prior (1998–2000) NTM isolation to identify incident isolation or disease. Index date was date of first positive culture for exposed patients (persons with NTM isolated) and was randomly assigned to potential unexposed controls. We used the RANUNI function in SAS ((https://support.sas.com/documentation/cdl/en/lrdict/64316/HTML/default/viewer.htm#a000202926.htm), which generates a number from 0 to 1. By using this number and the interval of the study period (January 1, 2001–December 31, 2013), a random integer was generated and added to the earliest start date of the study (January 1, 2001).

We used several data sources to characterize our cohort. To identify baseline underlying conditions, we used the Canadian Institute for Health Information Discharge Abstract Database (CIHI-DAD), which contains diagnostic and procedural information recorded during admissions to acute care hospitals, and the OHIP billing claims database, which contains physician billing claims for inpatient and outpatient services. Underlying conditions were identified by using validated algorithms, provincial registries, or in their absence, using diagnoses specified in either physician billing claims data or hospital discharge data (CIHI-DAD). Overall health status was estimated according to the adjusted clinical group case-mix system based on diagnostic information from health services use in the 2 years before the index date (*2*). Healthcare utilization (hospitalizations and emergency department visits) was assessed by using the CIHI-DAD and the CIHI National Ambulatory Care Reporting System. Income was estimated according to Statistics Canada median incomes of postal code regions. Rurality versus urbanity was quantified according to the Rural Index of Ontario grouping (*3*).

Validated algorithms were available for identifying persons with asthma, chronic kidney disease/end stage renal disease (*4*) (chronic kidney disease and end stage renal disease combined as a single variable for analyses), chronic obstructive pulmonary disease (*5*,*6*), diabetes mellitus (*7*), gastresophageal reflux disease (*8*), HIV infection (*9*), and rheumatoid arthritis (*10*,*11*) before the index date. Provincial registries were available and used for identifying lung malignancies and solid organ transplantation. Public Health Ontario Laboratory Services microbiology data were used to identify prior tuberculosis. For underlying conditions lacking validated algorithms and without available provincial registries, we used diagnoses specified in either physician billing claims data or CIHI-DAD. Bronchiectasis was defined by $\geq$1 physician billing claim and/or $\geq$1 hospital discharges with a diagnosis of bronchiectasis in accordance with the following codes: 494 (OHIP and International Classification of Diseases, Ninth Revision [ICD-9] codes) or J47, Q33.4, Q89.3 (ICD-10-CA codes) from April 1, 1991, to the index date. Cystic fibrosis was defined by $\geq$1 hospitalization with either of ICD-9 diagnosis of 277.0 or ICD-10 diagnosis of E84.X from April 1, 1991, to the index date. Hematopoietic stem cell transplantation was defined by procedure codes for transfusion of homologous/autologous stem cells, identified from CIHI-DAD and physician billing claims. Interstitial lung disease was defined by $\geq$1 physician billing claim and/or $\geq$1 hospital discharges with a diagnosis of interstitial lung disease in accordance

with the following codes: 515 (OHIP and ICD-9 codes) or J84, J84.0-J84.9 (ICD-10-CA codes) from April 1, 1991, to the index date.

Statistical Analysis

Multivariable logistic regression was used to compute 12 propensity scores, 1 for each species–condition combination of interest, comprising 6 individual species/species group (*M. avium* complex, *M. xenopi*, *M. fortuitum*, M. *abscessus*, *M. kansasii*, and “other species”) according to the conditions (NTM-PI and NTM-PD). Propensity scores estimated the patient-level likelihood of having the species-disease combinations, using all variables listed in Technical Appendix Table 1, with the exception of age and sex (used subsequently for matching), and HIV infection, solid organ or stem cell transplantation, cystic fibrosis, and prior tuberculosis. Initial inclusion of these latter low-frequency variables in propensity score calculations led to substantially fewer NTM patients successfully matching to an unexposed person. We matched each patient with NTM (exposed person) to an Ontario resident without NTM (unexposed control) who shared the age (years), sex, and index date (± 90 days) and had a propensity score value within 0.2 × SD of the exposed patient (*12*). Controls were selected without replacement, so 1 person could serve as an unexposed control for only 1 NTM patient.

We used Cox proportional hazards models to study survival; to account for the nonindependence of persons in the matched pairs, we used models with robust standard errors (or robust sandwich estimates) (*13*). The low-frequency variables not included in the propensity score (Tech App Table 1) were explored for inclusion as covariates in survival analyses comparing propensity-matched groups, using the Hosmer-Lemeshow approach (i.e., retained if including the covariate changed the risk estimate by $\geq$10%) (*14*). None of the covariates significantly affected the results and so were not included in the final models.

To study whether survival was affected by NTM-PD with >1 NTM species (multispecies NTM-PD), we compared the survival of NTM-PD patients infected with 1 species with patients who fulfilled criteria for >1 species. Persons who acquired NTM-PD of another species on or during follow-up of initial NTM-PD were considered to have multispecies NTM-PD. Initially, persons with multispecies NTM-PD appeared to survive longer than persons with single-species NTM-PD. However, after comparing survival curves of multispecies and single-species NTM-

PD, it appeared that the assumption of proportional hazards was violated. Time to second NTM species infection appeared to inflate survival of patients with multispecies NTM-PD, a form of immortal time bias (*15*). Consequently, for survival analyses of single-species versus multispecies NTM-PD, we modeled status of single-species versus multispecies NTM as a time-varying covariate to address this issue. Comparisons between groups that were not propensity-matched (NTM-PD vs. NTM-PI, and single-species vs. multispecies NTM-PD) included all Ontarians with NTM species–conditions of interest and were adjusted for age, sex, and covariates used to characterize our cohort.

Secondary survival analyses were performed excluding patients who died within 30 days after their NTM index date, based on the assumption that death so soon after NTM index date was unlikely to be related to the NTM condition. Analyses were performed by using SAS Enterprise Guide, version 6.1 (SAS Institute, Cary, NC, USA). All tests were 2-tailed with the type 1 error (α) rate set at 5%.

Results

During the 13-year study period, 20,617 Ontarians had incident NTM isolation from respiratory tract specimens, comprising 10,936 (53%) with NTM-PI and 9,681 (47%) with NTM-PD. Propensity score matching was successful for 9,967/10,936 (91%) of NTM-PI patients and 8,469/9,681 (87%) of NTM-PD patients. Differences between matched and unmatched patients are presented in Technical Appendix Table 1. Compared with matched patients, patients who could not be matched to unexposed controls were older (NTM-PI median 74 vs. 64 years, $p<0.001$ and NTM-PD median 72 vs. 70 years, $p<0.001$) and had higher frequencies of underlying conditions with higher mean adjusted clinical group case mix (*2*) numbers (NTM-PI 12.6 vs. 8.9, $p<0.001$ and NTM-PD 12.4 vs. 9.8, $p<0.001$). In addition, unmatched patients, compared with matched patients, had substantially reduced survival at 1 year (76.1% vs. 91.0%, respectively) and 5 years (46.3% vs. 76.0%, respectively) for all NTM-PI, and at 1 year (75.3% vs. 85.7%, respectively) and 5 years (47.7% vs. 65.4%, respectively) for all NTM-PD.

References

1. Griffith DE, Aksamit T, Brown-Elliott BA, Catanzaro A, Daley C, Gordin F, et al.; ATS Mycobacterial Diseases Subcommittee; American Thoracic Society; Infectious Disease Society of America. An official ATS/IDSA statement: diagnosis, treatment, and prevention of nontuberculous mycobacterial diseases. Am J Respir Crit Care Med. 2007;175:367–416. Erratum in: Am J Respir Crit Care Med. 2007;175:744–5. PubMed http://dx.doi.org/10.1164/rccm.200604-571ST

2. Reid RJ, MacWilliam L, Verhulst L, Roos N, Atkinson M. Performance of the ACG case-mix system in two Canadian provinces. Med Care. 2001;39:86–99. PubMed http://dx.doi.org/10.1097/00005650-200101000-00010

3. Kralj, B. Measuring "rurality" for purposes of health-care planning: an empirical measure for Ontario. Ontario Medical Review. 2000;67(10):33–42.

4. Vlasschaert ME, Bejaimal SA, Hackam DG, Quinn R, Cuerden MS, Oliver MJ, et al. Validity of administrative database coding for kidney disease: a systematic review. Am J Kidney Dis. 2011;57:29–43. PubMed http://dx.doi.org/10.1053/j.ajkd.2010.08.031

5. Gershon AS, Wang C, Guan J, Vasilevska-Ristovska J, Cicutto L, To T. Identifying individuals with physcian diagnosed COPD in health administrative databases. COPD. 2009;6:388–94. PubMed http://dx.doi.org/10.1080/15412550903140865

6. Gershon AS, Wang C, Guan J, Vasilevska-Ristovska J, Cicutto L, To T. Identifying patients with physician-diagnosed asthma in health administrative databases. Can Respir J. 2009;16:183–8. PubMed http://dx.doi.org/10.1155/2009/963098

7. Hux JE, Ivis F, Flintoft V, Bica A. Diabetes in Ontario: determination of prevalence and incidence using a validated administrative data algorithm. Diabetes Care. 2002;25:512–6. PubMed http://dx.doi.org/10.2337/diacare.25.3.512

8. Lopushinsky SR, Covarrubia KA, Rabeneck L, Austin PC, Urbach DR. Accuracy of administrative health data for the diagnosis of upper gastrointestinal diseases. Surg Endosc. 2007;21:1733–7. PubMed http://dx.doi.org/10.1007/s00464-006-9136-1

9. Antoniou T, Zagorski B, Loutfy MR, Strike C, Glazier RH. Validation of case-finding algorithms derived from administrative data for identifying adults living with human immunodeficiency virus infection. PLoS One. 2011;6:e21748. PubMed http://dx.doi.org/10.1371/journal.pone.0021748

10. Widdifield J, Bernatsky S, Paterson JM, Tu K, Ng R, Thorne JC, et al. Accuracy of Canadian health administrative databases in identifying patients with rheumatoid arthritis: a validation study using the medical records of rheumatologists. Arthritis Care Res (Hoboken). 2013;65:1582–91. PubMed

11. Widdifield J, Bombardier C, Bernatsky S, Paterson JM, Green D, Young J, et al. An administrative data validation study of the accuracy of algorithms for identifying rheumatoid arthritis: the influence of the reference standard on algorithm performance. BMC Muskuloskeletal Disorders. 2014;15(216).

12. Austin PC. Optimal caliper widths for propensity-score matching when estimating differences in means and differences in proportions in observational studies. Pharm Stat. 2011;10:150–61. PubMed http://dx.doi.org/10.1002/pst.433

13. Lu SE, Wang MC. Cohort case-control design and analysis for clustered failure-time data. Biometrics. 2002;58:764–72. PubMed http://dx.doi.org/10.1111/j.0006-341X.2002.00764.x

14. Hosmer DW, Lemeshow S, Sturdivant RX. Model building strategies and methods for logistic regression. In: Applied logistic regression. 3rd ed. Hoboken (NJ): John Wiley & Sons; 2013. pp. 89–152.

15. Jones M, Fowler R. Immortal time bias in observational studies of time-to-event outcomes. J Crit Care. 2016;36:195–9. PubMed http://dx.doi.org/10.1016/j.jcrc.2016.07.017

16. Austin PC, Grootendorst P, Anderson GM. A comparison of the ability of different propensity score models to balance measured variables between treated and untreated subjects: a Monte Carlo study. Stat Med. 2007;26:734–53. PubMed http://dx.doi.org/10.1002/sim.2580

Technical Appendix Table 1. Characteristics of patients with NTM pulmonary disease and pulmonary infection, according to whether they were successfully matched to unexposed controls, Ontario, Canada, 2001–2013*

	Pulmonary disease, N = 9,681			Pulmonary infection, N = 10,936		
Characteristic	Matched, n = 8,469	Unmatched, n = 1,212	SDM	Matched, n = 9,967	Unmatched, n = 969	SDM
Female sex, %	50.6	52.6	0.04	48.6	45.0	0.07
Median age, y (IQR)	70 (57–78)	72 (60–80)	0.15	64 (48–76)	74 (62–81)	0.52
Income quintile, %†						
1 (lowest)	26.6	27.0	0.01	30.8	36.9	0.13
2	21.4	23.5	0.05	23.0	22.3	0.02
3	18.0	17.5	0.01	17.2	14.9	0.06
4	16.4	15.2	0.03	14.9	13.1	0.05
5	17.2	16.3	0.02	13.5	11.6	0.06
Residential setting, %‡						
Rural	2.9	1.4	0.1	2.4	0.9	0.11
Urban	88.9	93.2	0.15	92.2	96.3	0.18
Suburban	8.2	5.4	0.11	5.4	2.8	0.13
Underlying condition, %						
Asthma	32.3	54.6	0.46	29.2	61.0	0.67
COPD	45.7	90.1	1.08	35.7	91.7	1.43
Diabetes	19.3	24.2	0.12	18.6	26.5	0.19
Rheumatoid arthritis	3.1	6.0	0.14	2.7	6.1	0.17
Chronic kidney disease	7.5	12.2	0.16	6.2	14.1	0.27
Gastresophageal reflux disease	16.5	22.8	0.16	14.6	23.2	0.22
Bronchiectasis	8.5	53.4	1.11	6.1	55.9	1.28
Interstitial lung disease	4.9	30.4	0.71	3.3	27.7	0.72
Lung cancer	5.1	27.6	0.64	2.3	12.2	0.39
HIV infection§	1.9	0.6	0.12	1.7	2.2	0.03
Solid organ transplantation§	0.7	6.1	0.3	0.4	1.8	0.13
BMT§	0.6	0.7	0.02	0.2	0.8	0.1
Cystic fibrosis§	0.6	3.3	0.19	0.5	1.8	0.12
Prior tuberculosis§	1.9	0.9	0.08	3.0	2.0	0.07
Hospitalizations, mean ± SD¶	0.34 ± 0.79	0.92 ± 1.51	0.48	0.31 ± 0.77	1.10 ± 1.60	0.63
ED visits, mean ± SD¶	0.85 ± 1.16	1.44 ± 1.61	0.42	0.80 ± 1.20	1.83 ± 1.96	0.63
ACG diagnoses, mean ± SD	9.79 ± 3.89	12.40 ± 3.34	0.72	8.94 ± 4.15	12.63 ± 3.25	0.99

*Matching performed according to age (years), sex, index date (± 90 d), and propensity score (estimating the patient-level likelihood of species-specific NTM pulmonary disease) value within 0.2 × SD of the exposed patient. ACG, adjusted clinical group using the ACG case mix system (*2*); BMT, hematopoietic stem cell transplantation; COPD, chronic obstructive pulmonary disease; ED, emergency department; IQR, interquartile range; NTM, nontuberculous mycobacteria; SDM, standardized difference of the mean (value <0.1 generally considered not significant) (*16*);
†Total numbers may not add to 100% because of rounding and missing income data in 0.4% with disease and 0.2% of controls
‡Residential setting characterized by Rural Index of Ontario (*3*).
§Baseline characteristics not included in the propensity score due to their effect to substantially reduce successful matching of exposed cases with unexposed controls; Inclusion of these variables as covariates was explored, but none significantly altered the HR point estimates
¶Number of events (mean ± SD) in year before index date.

Technical Appendix Table 2. Income and rurality of patients with NTM pulmonary disease and isolation and matched persons without NTM–MAC, *Mycobacterium xenopi*, and *M. abscessus*, Ontario, Canada, 2001–2013*

	MAC			*M. xenopi*			*M. abscessus*		
Characteristic	Exposed	Control	SDM	Exposed	Control	SDM	Exposed	Control	SDM
Disease	n = 5,543	n = 5,543		n = 1,975	n = 1,975		n = 201	n = 201	
Income quintile, %†									
1 (lowest)	27	25	0.04	26	23	0.07	19	14	0.13
2	21	22	0.02	22	23	0.02	16	22	0.14
3	18	18	0	17	19	0.04	25	17	0.21
4	16	16	0.01	17	17	0.01	18	23	0.14
5	18	18	0.02	17	19	0.04	21	23	0.06
Residential setting, %‡									
Rural	3	4	0.03	2	3	0.05	5	7	0.09
Urban	89	86	0.07	93	92	0.04	83	81	0.04
Suburban	8	10	0.06	5	5	0.01	13	12	0.01
Isolation	n = 5,242	n = 5,242		n = 2,693	n = 2,693		n = 162	n = 162	
Income quintile, %†									
1 (lowest)	31	31	0.01	31	27	0.07	24	17	0.17
2	23	23	0.02	22	25	0.08	22	24	0.04
3	17	17	0.01	17	16	0.01	24	19	0.11
4	15	16	0.03	16	17	0.03	15	17	0.05
5	13	14	0.02	15	14	0.01	15	23	0.19
Residential setting, %‡									
Rural	3	3	0.03	1	2	0.05	4	4	0
Urban	92	90	0.06	96	94	0.06	88	87	0.02
Suburban	6	7	0.06	3	4	0.04	8	9	0.02

*Matching performed according to age (years), sex, index date (± 90 d), and propensity score (estimating the patient-level likelihood of species-specific NTM pulmonary disease or isolation) value within 0.2 × SD of the exposed patient, Disease was defined as NTM pulmonary disease defined by the presence of >1 positive sputum sample for the same species or 1 positive bronchoscopic or biopsy specimen.
Isolation was defined as NTM pulmonary isolation defined by the presence of 1 positive sputum specimen. Controls were persons without NTM matched by age, sex, index date and propensity score. MAC, Mycobacterium avium complex; NTM, nontuberculous mycobacteria; SDM, standardized difference of the mean (value of <0.1 generally considered not significant) (*16*).
†Total numbers may not total 100% because of rounding and missing income data in 0.4% of patients with disease and 0.2% of controls.
‡Residential setting characterized by Rural Index of Ontario (*3*).

Technical Appendix Table 3. Characteristics of patients with NTM pulmonary disease and matched persons without NTM–*Mycobacterium fortuitum*, *M. kansasii*, and other species, Ontario, Canada, 2001–2013*

	M. fortuitum			*M. kansasii*			Other species		
Characteristic	Disease, n = 236	Control, n = 236	SDM	Disease, n = 144	Control, n = 144	SDM	Disease, n = 370	Control, n = 370	SDM
Female sex, %	45	45	0	34	34	0	51	51	0
Median age, y (IQR)	66 (53–76)	66 (53–76)	0	64 (50–73)	64 (50–73)	0	67 (54–76)	67 (54–76)	0
Income quintile, %†									
1 (lowest)	36	36	0	30	35	0.12	23	20	0.07
2	19%	19	0.01	27	24	0.08	23	23	0
3	17	15	0.06	16	18	0.06	17	16	0.03
4	19	22	0.07	14	10	0.11	22	21	0.01
5	9	8	0.05	13	13	0.02	15	20	0.12
Residential setting, %‡									
Rural	0–2§	0%–2†	0	0%–4§	0%–4§	0.07	5	4	0.05
Urban	87–89§	85%–87§	0.07	79%–83§	79%–82	0	81	84	0.07
Suburban	11	13	0.08	17	18	0.02	13	12	0.05
Underlying condition, %									
Asthma	32	26	0.13	31	28	0.08	32	25	0.15
COPD	39	47	0.17	52	58	0.13	39	44	0.11
Diabetes	23	23	0	13	17	0.12	24	29	0.12
Rheumatoid arthritis	4	<3§	0.16	<4§	<4§	0.12	4	4	0.01
Chronic kidney disease	8	6	0.1	9	10	0.02	8	9	0.03
GERD	15	17	0.07	13	15	0.06	19	20	0.03
Bronchiectasis	7	3	0.2	8	4	0.15	7	5	0.1
Interstitial lung disease	5	<3§	0.14	<4§	<4§	0	6	6	0.01
Lung cancer	4	<3§	0.13	6	<4§	0.18	5	3	0.11
HIV infection¶	<3§	0	0.13	9	<4§	0.39	<2§	0	0.15
Solid organ transplantation¶	<3§	0	0.09	<4§	0	0.12	<2§	<2§	0.07
BMT¶	<3§	0	0.16	0	0	.	<2§	0	0.1
Cystic fibrosis¶	<3§	0	0.09	<4§	0	0.12	<2§	0	0.1
Prior tuberculosis¶	3	0	0.26	0	0	.	2	0	0.22
Hospitalizations, mean ± SD #	0.37 ± 0.81	0.33 ± 0.79	0.05	0.49 ± 0.92	0.34 ± 1.10	0.14	0.43 ± 0.86	0.35 ± 0.82	0.09
ED visits, mean ± SD #	0.82 ± 1.22	0.81 ± 1.60	0.01	1.19 ± 1.26	0.85 ± 2.10	0.19	0.86 ± 1.19	0.91 ± 1.70	0.04
ACG diagnoses, mean ± SD	9.5 ± 3.9	9.2 ± 3.6	0.07	9.7 ± 3.7	9.4 ± 4.1	0.08	9.9 ± 4.2	9.6 ± 4.1	0.08

*Matching performed according to age (years), sex, index date (± 90 d), and propensity score (estimating the patient-level likelihood of species-specific NTM pulmonary disease) value within 0.2 × SD of the exposed patient. Controls were persons without NTM matched by age, sex, index date and propensity score. NTM pulmonary disease was defined by the presence of >1 positive sputum for the same species or 1 positive bronchoscopic or biopsy specimen. ACG, adjusted clinical group diagnoses using the ACG case mix system (*2*); BMT, hematopoietic stem cell transplant; ED, emergency department; GERD, gastresophageal reflux disease; IQR, interquartile range; NTM, nontuberculous mycobacterium; SDM, standardized difference of the mean (value of <0.1 generally considered not significant) (*16*).
†Total numbers might not total 100% because of rounding and missing income data in 0.4% with disease and 0.2% of controls.
‡Residential setting characterized by Rural Index of Ontario (*3*).
§Range reported because of small cell size (direct or by inference), which according to privacy regulations cannot be reported.
¶Baseline characteristics not included in the propensity score because of their effect to substantially reduce successful matching of exposed cases with unexposed controls; Inclusion of these variables as covariates was explored, but none significantly altered the hazard ratio point estimates.
#Number of events (mean ± SD) in year before index date.

Technical Appendix Table 4. Characteristics of patients with NTM pulmonary isolation and matched individuals without NTM - *Mycobacterium fortuitum, M. kansasii*, and other species, Ontario, Canada, 2001–2013*

Characteristic	*M. fortuitum*			*M. kansasii*			Other species		
	Isolation, n = 654	Control, n = 654	SDM	Isolation, n = 92	Control, n = 92	SDM	Isolation, n = 1,124	Control, n = 1,124	SDM
Female sex, %	43	43	0	32	32	0	46	46	0
Median age, y (IQR)	63 (45–74)	63 (45–74)	0	66 (51–78)	66 (51–78)	0	63 (48–75)	63 (48–75)	0
Income quintile, %†									
1 (lowest)	35	36	0.03	24	27	0.07	28	27	0.03
2	24	24	0	21	22	0.03	24	26	0.03
3	17	16	0.01	24	23	0.03	19	18	0.01
4	13	13	0.01	16	15	0.03	16	16	0
5	11	11	0.02	15	13	0.06	13	13	0
Residential setting, %‡									
Rural	4	4	0.04	<6†	0	0.21	4	5	0.07
Urban	90	87	0.08	79–85§	80	0.06	88	83	0.13
Suburban	7	9	0.07	15	20	0.11	9	12	0.11
Underlying condition, %									
Asthma	31	25	0.13	29	27	0.05	26	22	0.09
COPD	31	34	0.05	48	57	0.17	33	37	0.09
Diabetes	20	25	0.11	17	14	0.09	19	21	0.04
Rheumatoid arthritis	2	2	0.01	<6§	0	0.26	2	2	0.04
Chronic kidney disease	5	5	0.01	<6§	<6§	0.15	6	7	0.04
GERD	14	14	0.01	20	17	0.06	16	16	0
Bronchiectasis	5	5	0.01	10	<6§	0.27	5	5	0.01
Interstitial lung disease	3	2	0.09	<6§	<6§	0.07	4	3	0.06
Lung cancer	3	2	0.03	<6§	7	0.15	2	2	0.05
HIV infection¶	1	<1§	0.14	<6§	<6§	0.25	2	<1§	0.15
Solid organ transplantation¶	<1§	<1§	0.03	0	0		1	0	0.13
BMT¶	<1§	0	0.08	0	0		<1§	<1§	0.06
Cystic fibrosis¶	1	<1§	0.1	0	0	.	<1§	0	0.09
Prior tuberculosis¶	3	0	0.24	<6§	0	0.15	4	<1§	0.27
Hospitalizations, mean ± SD#	0.30 ± 0.89	0.25 ± 0.66	0.06	0.45 ± 0.80	0.18 ± 0.42	0.41	0.28 ± 0.67	0.26 ± 0.72	0.03
ED visits, mean ± SD#	0.71 ± 1.27	0.66 ± 1.31	0.04	1.21 ± 1.40	0.57 ± 0.87	0.55	0.70 ± 1.07	0.73 ± 1.51	0.02
ACG diagnoses, mean ± SD	8.5 ± 4.3	8.2 ± 4.0	0.06	9.3 ± 4.5	7.9 ± 3.9	0.33	8.7 ± 4.1	8.4 ± 4.0	0.07

*Matching performed according to age (years), sex, index date (± 90 d), and propensity score (estimating the patient-level likelihood of species-specific NTM pulmonary isolation) value within 0.2 × SD of the exposed patient. Controls were persons without NTM matched by age, sex, index date, and propensity score. Isolation was defined as NTM pulmonary isolation defined by the presence of 1 positive sputum specimen the same species or 1 positive bronchoscopic or biopsy specimen. ACG ; adjusted clinical group diagnoses using the ACG case mix system (*2*); BMT, hematopoietic stem cell transplant; COPD, chronic obstructive pulmonary disease; ED, emergency department; IQR, interquartile range; NTM, nontuberculous mycobacteria; SDM, standardized difference of the mean (value of <0.1 generally considered not significant) (*16*); empty cells indicate value undefined.

†Total numbers may not total 100% because of rounding and missing income data in 0.5% with isolation and 0.4% of controls.

‡Residential setting characterized by Rural Index of Ontario (*3*)

§Range reported because of small cell size (direct or by inference), which according to privacy regulations cannot be reported

¶Baseline characteristics not included in the propensity score due to their effect to substantially reduce successful matching of exposed cases with unexposed controls; Inclusion of these variables as covariates was explored, but none significantly altered the HR point estimates

#Number of events in year before index date.
